# Four questions to predict cognitive decline in de novo Parkinson’s disease

**DOI:** 10.1038/s41531-025-00958-5

**Published:** 2025-04-25

**Authors:** Jan Hlavnička, Josef Mana, Ondrej Bezdicek, Martin Čihák, Filip Havlík, Dominik Škrabal, Tereza Bartošová, Karel Šonka, Evžen Růžička, Petr Dušek

**Affiliations:** 1 Department of Neurology and Centre of Clinical Neuroscience, First Faculty of Medicine, Charles University and General University Hospital, Prague, Czechia; 2 Department of Circuit Theory, Faculty of Electrical Engineering, Czech Technical University in Prague, Prague, Czechia

**Keywords:** Risk factors, Parkinson's disease

## Abstract

Early identification of cognitive decline (CD) in de novo Parkinson’s disease (PD) is crucial for choosing appropriate therapies and recruiting for clinical trials. However, existing prognostic models lack flexibility, scalability and require costly instrumentation. This study explores the utility of standard clinical questionnaires and criteria to predict CD in de novo PD. A total of 186 patients from the Parkinson Progression Markers Initiative (PPMI) and 48 patients from the Biomarkers of Parkinson’s Disease project (BIO-PD) underwent clinical interviews, comprehensive tests, and questionnaires. A model based only on age of disease onset, history of stroke, history of fainting, and vocalization during dreams predicted CD in 2 and 4-year horizons with an area under curve (AUC) of 70% ± 10% standard deviation (cross-validated PPMI), 79% (overall PPMI), and 78% (validation in BIO-PD). This approach enables rapid preliminary screening using just four simple questions, achieving predictive accuracy comparable to instrumentation-based methods while reducing assessment time.

## Introduction

Cognitive impairment and dementia are common in Parkinson’s disease (PD). Impaired cognition was reported in 20–40% of early untreated PD and 75% of PD patients with disease duration above 10 years^1–6^. PD patients with mild cognitive impairment (MCI) are at increased risk of developing dementia, and MCI is believed to represent a transitioning stage into PD dementia^7–9^ characterized by predominant executive dysfunction, i.e., the inability to plan, organize, and regulate goal-directed behavior^10–12^. Cognitive dysfunction may reduce quality of life more than motor disturbances. It is associated with higher mortality rates^8,13,14^, worsening of health-related quality of life^15^, and places an increased burden on caregivers^16^. Therefore, identification, prevention, and treatment of cognitive decline in PD play a key role in maintaining and/or improving patients’ quality of life, increasing life expectancy, and reducing healthcare costs^8^.

The main clinical risk factors of PD dementia include advanced age at disease onset, long disease duration, smoking, early occurrence of confusion or psychotic symptoms related to dopaminergic treatment, predominant speech, gait and axial involvement, severe motor symptoms, poor performance in the cognitive test at baseline, and non-motor symptoms such as depression, anxiety, orthostatic hypotension, and rapid eye movement sleep behavior disorder (RBD)^17–22^. Additional promising predictors are pathogenic genetic variants in *apolipoprotein E*, *microtubule-associated protein Tau*, *α‑Synuclein*, and *glucocerebrosidase* genes, decreased cerebrospinal fluid levels of amyloid-β_42_, and abnormal electroencephalogram^23–25^.

Identification of patients at risk of cognitive decline by monitoring the evolution of scores of cognitive tests in time can be difficult due to cognitive fluctuations caused by many factors and the requirements to perform standardized follow-up cognitive tests. A more desirable approach is to determine risk by analyzing demographic and clinical data via complex algorithms and intervening early by non-pharmacological approaches such as engagement in cognitive and social activities and physical exercises^26,27^.

The need for an integrated and widely applicable prognostic model of cognitive decline in PD that could help clinicians make informed decisions for preventative care, akin to the user-friendly Framingham risk score for assessing the risk of developing coronary heart disease, has been highlighted by previous studies^25,28,29^. While the computational models based on age, non-motor assessments, dopamine transporter imaging, cerebrospinal fluid biomarkers, and genetic testing showed considerable accuracies, with an area under curve ranging between 0.8 and 0.85, their potential for routine clinical use is constrained^25,28,29^.

Further development of prognostic models and their translation into clinical practice is hindered by the limited sample size of existing cohorts and the lack of independent datasets for blind evaluation. Testing without independent data may yield overly optimistic results, especially when a complex model is trained and tested with a large set of features. Transparent knowledge-driven criteria expressed as likelihood ratios and combined via Bayesian naïve classifier are preferable in such a setting^30^. Moreover, limiting the model to the standard battery of tests, such as well-established questionnaires, might reduce the cost of screening and consequently increase its availability.

Considering the expected rapid increase in the prevalence of PD in low-income countries, medical care inequalities across the globe^31^, and missing data on prevalence, management, societal burden, and effective risk prediction in these regions^27^; having a widely applicable prognostic model that relies only on answers or scores gathered during the standard neurological examination could have a major impact on the medical care of patients with PD.

To our knowledge, no such transparent prognostic model of cognitive decline that can be refined, shared, or extended easily by new clinical features has been presented with a blind test to date. This study introduces a simple, quick, effective solution for predicting cognitive decline that can be further extended, updated, and delineated as new information becomes available. Moreover, the prediction can be carried out with a pen-and-paper or an online calculator (http://araw.mede.uic.edu/cgi-bin/testcalc.pl) using only the data gathered within the scope of standard clinical examination and questionnaires. This model can thus improve prevention, diagnosis, and treatment of cognitive impairment in PD patients, even in non-equipped health-care centers.

## Results

### Progression of cognitive decline

**Fig. 1:**
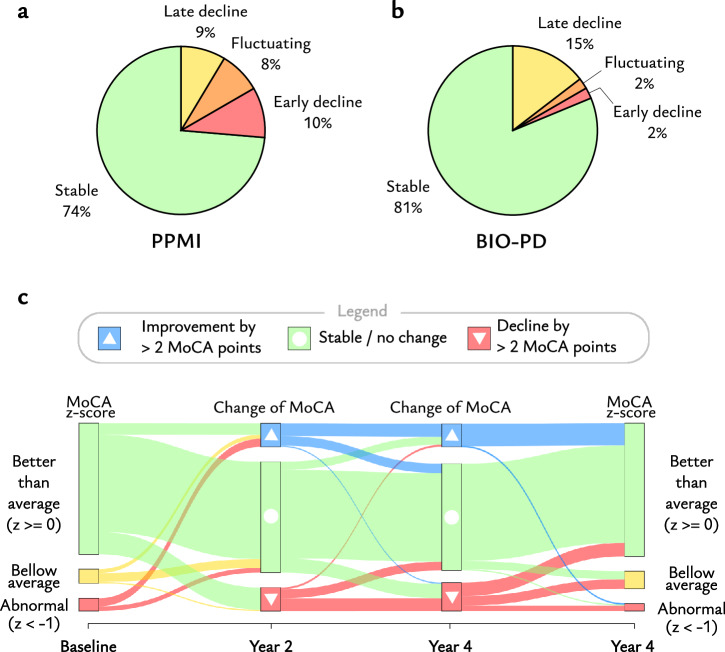
Longitudinal Evolution of Cognitive Function in Both Study Cohorts. Pie charts illustrating proportions of patients with stable cognitive performance (no change between baseline and follow-ups for more than 2 points of MoCA), early decline (decrease of MoCA between baseline and 2nd year higher than 2 points without improvement in the second follow-up), late decline (decrease of MoCA only between baseline and 4th year higher than 2 points), and fluctuations (decline in 2nd year followed by improvement, so that change in MoCA observed between baseline and 4th year is lower than 2 points) is plotted for PPMI (**a**) and BIO-PD data (**b**). Note that the percentages were rounded to whole numbers. Sankey diagram (**c**) visualizes the distribution of normalized MoCA in baseline and 4th year, as well as changes of absolute MoCA scores between follow-up visits for both PPMI and BIO-PD databases combined. The cognitive decline was determined by a decrease of the MoCA score by more than 2 points, whereas the improvement was determined by an increase of the MoCA score by more than 2 points. All other changes were considered stable. All subjects with MoCA z-score above 0 were considered as above average, all subjects between -1 z-score and 0 as below average, and all subjects below -1 z-score as abnormal for baseline and 4th year follow-up. The height of all boxes within each column is proportional to number of subjects in corresponding sub-groups. PPMI Parkinson Progression Markers Initiative, BIO-PD Biomarkers of Parkinson’s disease project, MoCA Montreal Cognitive Assessment.

A total of 26% of patients in the PPMI database and 19% of patients in the BIO-PD database showed a cognitive decline in the 2nd and/or 4th-year visit. The majority of patients (78%) with cognitive decline in the BIO-PD database showed late cognitive decline in the 4th year follow-up. In contrast, early cognitive decline in the 2nd year follow-up was observed in 37%, and late cognitive decline in 33% of patients with cognitive decline in the PPMI database, with comparable frequencies (Fig. 1a, b). The complicated nature of changes in MoCA score is described in Supplementary Information: Heterogeneity in the Progression of Cognitive Changes and illustrated in Fig. 1c.

### Prior probabilities of cognitive decline

The prior probabilities of cognitive dysfunction within PD age-of-onset brackets were increased for age of onset below 45 years. The probabilities above 45 years were relatively low and gradually increased with the age of onset. Note that the number of subjects was low for brackets at the lower and upper limits of the range, so we combined PPMI with BIO-PD data to obtain smooth estimates. Please see a summary in Table 1.

**Table 1:** Overview of the number of subjects and the prior probability of future cognitive decline within age brackets for PPMI and BIO-PD databases

	PPMI		BIO-PD		Combined	
Age of onset	Subjects total	Prior probability	Subjects total	Prior probability	Subjects total	Prior probability
30–40	4	0.50	5	0.2	9	0.33
40–45	6	0.33	1	0	7	0.29
45–50	25	0.12	6	0	31	0.10
50–55	24	0.13	6	0	27	0.11
55–60	31	0.16	10	0.3	41	0.20
60–65	37	0.27	6	0	43	0.23
65–70	34	0.44	6	0.17	40	0.40
70–75	13	0.31	6	0.33	19	0.32
75–100	8	0.62	3	0.67	11	0.64

*PPMI* Parkinson Progression Markers Initiative, *BIO-PD* Biomarkers of Parkinson’s Disease Project.

### Predictive factors of cognitive decline

A total of 22 features showed sufficient likelihood ratios and proportionality to predict future cognitive decline in the PPMI dataset. The only protective factors were normal UPSIT score (negative LR = 0.27), no nocturia (question 13 of SCOPA-AUT, negative LR = 0.22), and no incontinence in the previous month (question 12 of SCOPA-AUT, negative LR = 0.50). Risk factors included history of stroke, moderate or severe light-headedness on standing (MDS-UPDRS I, item 12), moderate or severe speech disorder (MDS-UPDRS II, items 1), moderate or severe chewing and swallowing (MDS-UPDRS II, items 3), moderate or severe tremor (MDS-UPDRS II, items 10), compound right-sided rigidity of MDS-UPDRS III, involuntary loss of stools (SCOPA-AUT, item 7), frequent urination and nocturia (SCOPA-AUT, item 12 and 13), fainting (SCOPA-AUT, item 16), total SCOPA-AUT score, feeling upset (STAI-X 1, question 6), feeling difficulties are piling up (STAI-X 2, questions 8), taking things hard (STAI-X 2, question 11), persistent rumination (STAI-X 2, question 18), total STAI-X 2 score, vocalization during dreams (RBDSQ, question 6.1), inability to name rhinoceros in a picture (MoCA), drawing clock numbers incorrectly (MoCA), low performance in visuospatial/executive domain (MoCA), and low MoCA normalized to z-score.

None of the examined factors showed predictive power for both positive and negative outcomes. Only UPSIT, compound right-sided rigidity (MDS UPDRS III), history of stroke, history of fainting (item 16 of SCOPA-AUT), drawing clock numbers incorrectly (MoCA), low performance in visuospatial/executive domain (MoCA), and vocalization during dreams (question 6.1 of RBDSQ) showed sufficient likelihood ratio in the identification of future cognitive decline for both databases. All subjects with a history of stroke also presented a cognitive decline in the future. Thus, their LR+ was infinite, and a posterior probability is one, which means that all PD patients with a history of stroke will most likely show a cognitive decline in the future. Please see Table 2 for an overview of effective features and Supplementary Data summarizing all results on both analyzed datasets including cut-off thresholds.

**Table 2:** Overview of features relevant for prognosis of cognitive decline in de novo PD

		PPMI				BIO-PD			
Symptom	Description	LR+	LR-	Prop.	p	LR+	LR-	Prop.	p
**UPSIT total**	**UPSIT score lower than 31 for females or 30 for males**	**1.21**	**0.27**	**0.82**	**	**1.16**	**0.48**	**0.79**	*NS*
MDSUPDRS 1.12	Moderate or severe light-headedness on standing	2.80	0.99	0.01	*NS*	N/A	1.00	0.00	*NS*
MDSUPDRS 2.1	Moderate or severe speech disorder	2.80	0.99	0.01	*NS*	N/A	1.00	0.00	*NS*
MDSUPDRS 2.3	Moderate or severe chewing and swallowing	2.80	0.99	0.01	*NS*	N/A	1.00	0.00	*NS*
MDSUPDRS 2.10	Moderate or severe tremor	2.80	0.99	0.01	*NS*	0.00	1.08	0.06	*NS*
**Right-sided rigidity**	Sum of right upper and lower limb MDS-UPDRS III score higher than 5 *	**8.39**	**0.95**	**0.02**	*NS*	**4.33**	**0.91**	**0.04**	*NS*
**Stroke** ^§^	History of ischemic or hemorrhagic stroke *	***Infimum***	**0.94**	**0.02**	*	***Infimum***	**0.89**	**0.02**	*NS*
SCOPA-AUT 7	In the past month, have you had involuntary loss of stools? **	6.99	0.91	0.04	*	0.00	1.03	0.02	*NS*
SCOPA-AUT 12	In the past month, have you had to pass urine again within 2 hours of the previous time?	1.13	0.50	0.82	*NS*	0.47	10.83	0.85	*NS*
SCOPA-AUT 13	In the past month, have you had to pass urine at night?	1.18	0.22	0.85	*	1.01	0.96	0.77	*NS*
**SCOPA-AUT 16** ^§^	In the past 6 months, have you fainted? *	**2.80**	**0.97**	**0.02**	***NS***	**4.33**	**0.91**	**0.04**	***NS***
SCOPA-AUT total	SCOPA-AUT score higher than 20 **	2.33	0.94	0.06	*NS*	0.00	1.03	0.02	*NS*
STAIX 1 Question 6	I feel upset, very much so *	2.80	0.97	0.02	*NS*	N/A	1.00	0.00	*NS*
STAIX 2 Question 8	I feel that difficulties are piling up in such a way that I cannot overcome them, almost always *	5.59	0.97	0.02	*NS*	N/A	1.00	0.00	*NS*
STAIX 2 Question 11	I am inclined to take things hard, almost always	2.80	0.97	0.02	*NS*	N/A	1.00	0.00	*NS*
STAIX 2 Question 18	I take disappointments so keenly that I cannot get them out of my mind, almost always	2.10	0.97	0.04	*NS*	N/A	1.00	0.00	*NS*
STAIX 2 total	Trait anxiety score higher than 50 ***	2.15	0.88	0.12	*NS*	0.00	1.18	0.13	*NS*
MoCA Rhino	What is the name of an animal in the picture (Rhinoceros)?	2.24	0.95	0.05	*NS*	N/A	1	0	*NS*
MoCA Clock numbers	Draw ten past eleven on the clock. Are the numbers on the drawing correct or wrong?	2.80	0.93	0.13	*NS*	2.88	0.84	0.23	*NS*
MoCA Visuospatial/executive	Total score of visuospatial/executive dimension less than 3	8.39	0.95	0.02	*NS*	4.33	0.91	0.04	*NS*
MoCA total z-score	Total MoCA score normalized to z-score lower than -1	2.80	0.99	0.01	*NS*	0.93	1.04	0.35	*NS*
**RBDSQ 6.1** ^§^	I have had the following phenomenon during my dreams: speaking, shouting, swearing, laughing loudly ***	**2.70**	**0.56**	**0.30**	***	**2.17**	**0.60**	**0.31**	***NS***

The consistent features are indicated with bold font. The final simplified model is marked by §.

*BIO-PD* Biomarkers of Parkinson’s Disease, *Infimum* infinite value because all subjects with the symptom were correctly identified, *LR+* positive likelihood ratio, *LR−* negative likelihood ratio, *MDS-UPDRS* MDS-Unified Parkinson’s Disease Rating Scale, *N/A* not available (no subject presented the symptom), *NS* not significant, *PPMI* Parkinson Progression Markers Initiative, *Prop.* proportion of subjects with the feature.

*p < 0.05, **p < 0.01, ***p < 0.001, probabilities in the second column denote that the feature has a significant contribution to the model’s performance estimated from the likelihood ratio test statistic on the PPMI dataset for feature selection procedure. The probabilities in the 6th and 10th column represent nonrandom associations between presence of feature and future cognitive decline determined via Fisher’s exact test.

The majority of these predictive features also showed a significant difference between subjects with and without cognitive decline in baseline characteristics and change at the first follow-up (Supplementary Information: Comparison of Characteristics in Subjects with and without Cognitive Decline).

### Classification experiment

**Fig. 2:**
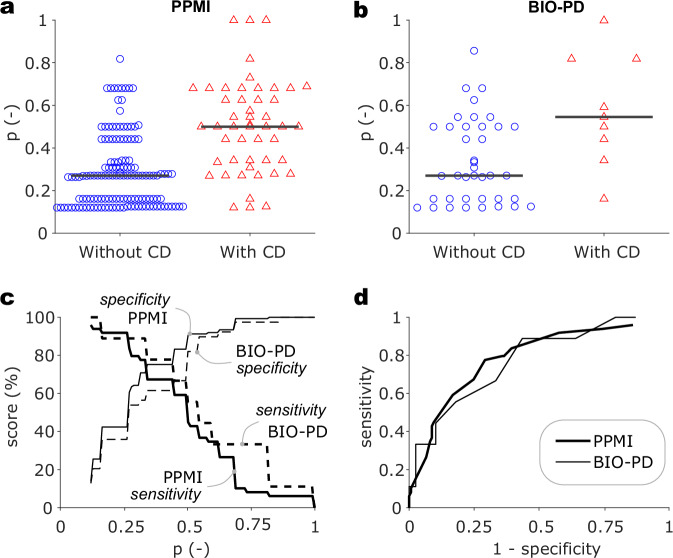
Predictive Performance of the Model. Dotplots represent the probability of cognitive decline determined by the predictive model on PPMI (**a**) and BIO-PD (**b**) datasets. MoCA progression equal to zero represents a group of subjects without any cognitive decline, whereas MoCA progression equal to 1 is the group showing the cognitive decline in at least one of the follow-up visits. Horizontal lines represent median. Sensitivity and specificity are illustrated in a single graph (**c**) regarding probability cut-offs for PPMI (thick solid line for sensitivity, thin solid line for specificity) and BIO-PD (thick dotted line for sensitivity, thin dotted line for specificity). The x-axis represents probability cut-off for which the score of sensitivity or specificity can be deduced on the corresponding line from *y*-axis. The receiver operating characteristics (**d**) are plotted for PPMI (thick solid line) and BIO-PD (thin solid line). The *x*-axis represents complementary value for specificity estimate on the whole range of probability cut-offs for which the corresponding sensitivity can be obtained on the *y*-axis. BIO-PD Biomarkers of Parkinson’s Disease, CD cognitive decline, PPMI Parkinson Progression Markers Initiative.

The cross-validation classification experiment on PPMI data demonstrated that the number of features can be further reduced without decreasing the effectiveness of the model. Therefore, we determined our model based on prior probability from age, history of stroke, history of fainting, and vocalization during dreams, considering that this information can be gathered easily during clinical interview via four simple questions and has strong underlying hypotheses. Using the prior probability combined from both datasets led to an AUC of 70% ± 10% standard deviation in cross-validated PPMI, 79% in overall PPMI data, and 78% in BIO-PD data. The performance did not improve when the model was updated with new information from the 1st year follow-up visit. Please see Fig. 2 depicting the performance and Table S1 in Supplementary Information for more details. The final model can be employed for a quick test via a graphical user interface called CogniPa, developed within the framework of this study^32^.

To evaluate the effectiveness of baseline neuropsychological screening in predicting future cognitive decline, we developed a predictor incorporating prior probability based on age, and MoCA items with the strongest predictive value—specifically, the visuospatial/executive subscore, the naming of a rhinoceros, and drawing numbers in the clock-drawing task. The model achieved an AUC of 61% ± 10% standard deviation in cross-validated PPMI data, 70% in the overall PPMI dataset, and 62% in BIO-PD data.

## Discussion

This study highlights the potential of non-instrumentational methods in predicting cognitive decline in de novo Parkinson’s disease. Rather than employing complex machine-learning models, we adopted traditional modeling with likelihood ratios, creating a practical, user-friendly prognostic model. Rigorously tested on blind data, the model based on age of disease onset, history of stroke, history of fainting, and vocalization during dreams demonstrated significant effectiveness and flexibility, enabling budget-friendly screening for cognitive decline without instrumentation.

Cognitive changes varied significantly across visits, with a minority (18% PPMI, 11% BIO-PD) showing a temporary decline in MoCA score below 26 points but returning to normal cognition. Identifying cognitive decline, considering a decrease in MoCA score between baseline and follow-up, appears thus more practical for early preventative interventions than applying a cutoff to the absolute baseline MoCA score (Supplementary Information: Heterogeneity in the Progression of Cognitive Changes). The effectiveness of cut-off values may be biased by age and education, as MoCA normalized to a z-score proved to be a strong predictor, while absolute MoCA did not. While the MoCA items for clock numbers and rhinoceros naming showed positive likelihood ratios, we remain skeptical about the predictive values of individual MoCA items, as no consistent pattern emerged within cognitive domains. Similarly, while the visuospatial/executive subscore had a high likelihood ratio in both datasets, its effectiveness fluctuated across different cut-off values, indicating an unacceptable level of noise. In fact, predictions based solely on age and MoCA were not particularly effective, highlighting the need for more in-depth cognitive assessment.

Although the UPSIT score was the only symptom showing a significant group difference between patients with and without a cognitive decline in both datasets, taking unimpaired olfaction as a protective factor was more favorable due to the great overlap between groups, considering the standard clinical cut-off. The previous studies on the PPMI dataset already reported an association between olfactory loss and future cognitive decline, hypothesizing a more severe extranigral pathology according to Braak staging^28,33,34^. Fullard et al. juxtaposed the findings of cerebrospinal fluid biomarkers such as tau, Aβ42, and tau/Aβ42 ratio, concluding that olfaction can serve as an independent proxy of distinct molecular pathology but its usefulness will require more validation^34^. Our findings on an independent dataset suggest that olfaction can indeed be an effective indicator of future cognitive decline, though its contribution to modeling may not be straightforward.

Of all the motor features, only compound right-sided rigidity showed sufficiently high effectiveness in predicting future cognitive decline in both datasets. The association between the rigid-akinetic motor subtype and cognitive decline is well aligned with previous research^35,36^. Also, right-sidedness has been linked to faster disease progression^35^. However, prior studies have reported inconsistent findings regarding the impact of motor symptom laterality on cognitive decline. While some studies, including ours, have found an association between right-sided symptom onset and cognitive decline^37^, others have reported either no effect or a more complex relationship^38^, with different cognitive profiles emerging in patients with right-versus left-sided symptom onset^38,39^. Theoretically, our findings may be attributed to the fact that MoCA is biased toward detecting left hemisphere impairment and is therefore more sensitive to cognitive decline in right-sided patients^40^.

The history of fainting in the last 6 months predicts future cognitive decline reliably in both datasets, though this symptom is not frequently present. Hypoperfusion of the brain may be the underlying factor of cognitive fluctuations or decline^19,20^. In addition to fainting, hypoperfusion has other manifestations such as feeling lightheaded or dizzy when standing, and previous research reported the association of these symptoms with cognitive dysfunction in PD for cross-sectional differences as well as longitudinal changes in MoCA scores^19^. Although the positive likelihood of light-headedness after standing for some time as assessed by question 15 of SCOPA-AUT was 1.72 and thus below the threshold, the BIO-PD data showed a relatively high value of 2.6, supporting that orthostatic symptoms might be indicative of future cognitive decline. Another interpretation of these findings is that orthostatic hypotension, and more generally autonomic dysfunctions, are associated with the diffuse-malignant or body-first PD subtypes, which lead to a more rapid disease progression with faster cognitive decline^41^.

Another autonomic symptom, involuntary loss of stools, was predictive in PPMI data; however, this symptom was rare in BIO-PD, and none of the affected individuals exhibited future cognitive decline. Nevertheless, previous research suggests a potential link between impaired gastrointestinal function and cognitive decline^42^. Finally, the relation between SCOPA-AUT and future cognitive decline in PPMI, but not in BIO-PD, highlights that while autonomic symptoms may play a role, they may lack consistency and frequency needed for reliable prediction of cognitive decline in early-stage PD.

Anxiety has previously been associated not only with PD-related cognitive decline but also with mild cognitive decline in older participants^21,43^. STAI-X scores predicted future cognitive decline effectively only in the PPMI dataset. STAI-X scores in BIO-PD data were too low to exceed the selected cutoffs, and no single patient in BIO-PD data answered Question 6 or Question 8 positively. We hypothesized that a cultural difference may influence the optimal cut-off, but a detailed analysis of the cut-off scores on the BIO-PD data was not convincing, and a specialized study will be required to answer this theory. The only STAI-X-based feature with a sufficient likelihood of 2.2 was question 6 from STAI-X1 with a cut-off value of 1. To conclude, anxiety could serve only as an additional feature to refine the prognosis, but its inconsistent association with cognitive evolution limits its applicability to build predictive models of cognitive decline.

The hallmark symptoms of the REM sleep behavior disorder, concentrated in question 6.1 of the RBDSQ: speaking, shouting, swearing, and laughing loudly during dreaming, proved to be both reliable and sufficiently common factor predicting future cognitive decline. Other characteristics covered by the RBDSQ questionnaire were rejected either due to lack of consistency of the threshold, such as the total score, or due to insignificant contribution to the final model. Though the presence of REM sleep behavior disorder is consistently linked to future cognitive decline^44^, some specific symptoms of REM sleep behavior may theoretically have increased predictive value.

A history of ischemic or hemorrhagic stroke was associated with future cognitive decline. A recent meta-analysis showed faster cognitive decline in non-PD persons with a history of stroke compared to the stroke-free population^45,46^. Given the consistent cognitive deterioration in our patients with a history of stroke, it is possible that the interaction between ischemic changes and α-synucleinopathy may contribute to this accelerated decline^46^. However, with the relatively low representation of stroke in our datasets (3 subjects in PPMI and 1 in BIOPD), we cannot draw definitive conclusions. We highlight this as a potential area for further investigation in larger cohorts of stroke patients with and without PD. The likelihood-ratio model allows stroke to be excluded from the final estimation by simply accounting for it as non-present.

Overall, the non-motor profile of predictors of future cognitive decline found in our study indicates a link to “body-first” or “diffuse-malignant” subtypes of PD, which both refer to an overlapping phenotype associated with REM sleep behavior disorder, orthostatic hypotension and other autonomic symptoms, and more rapid progression of neuropsychiatric symptoms and cognitive impairment^47–50^.

Of all the features, only the history of stroke, the history of fainting, and vocalization during dreams were consistently effective for both databases to constitute a minimum subset of predictors. A model based on these features and utilizing age of onset for calculation of prior probabilities was surprisingly effective for recognizing cognitive decline in both datasets, with an AUC of 70% ± 10% standard deviation in cross-validated PPMI and an AUC of 78% in the BIO-PD dataset. Such a performance is remarkable given that only four questions are needed to gather all the data, especially in the context of other models employing dozens of descriptors at substantial cost (Supplementary Information: Applicability of the Prognostic Model)^28^.

One of the main factors behind the performance is that the model was built as parsimonious as possible without the necessity to optimize parameters multidimensionally and by utilizing both knowledge and data for feature selection. Also, the likelihood ratios allow us to control the desired sensitivity and specificity by adjusting the p-value cut-off, which is advantageous over regressing future continuous MoCA. The prediction can also be interpreted as the upper boundary of future MoCA score by accounting for MoCA at the baseline and estimated change of 2 or more MoCA points for estimated cognitive decline.

The study targeted only de novo patients, which limits the applicability of the results to other conditions and PD stages. The obvious limitation of this study is that the severity of cognitive decline was not assessed in terms of clinical criteria for mild cognitive impairment or dementia in PD. Instead, we adopted well-established clinical thresholds of cognitive changes via MoCA scoring, allowing us to use two separate datasets for development and blind validation, thereby enhancing generalizability. Another limitation of this study is a relatively short prediction horizon of up to 4 years. Given the prevalence of dementia observed in the majority of patients (83%) over 20 years, likely influenced by age and multiple pathologies, the model should have accounted for factors emerging throughout the disease course for predicting outcomes over a longer duration^51^. Therefore, the limited timeframe allows for a simpler model that, however, is more advantageous, particularly in the context of identifying early deterioration and facilitating prompt rehabilitation initiatives. We scrutinized factors that could influence cognition, including education, history of depression and anxiety, diabetes, and history of intoxication or alcohol abuse. Among these, only education significantly influenced MoCA values. The impact of these factors on future changes in MoCA scores was found to be negligible. While we cannot rule out the influence of co-occurring conditions or factors outside the scope of our dataset, we are confident that the most relevant known factors do not bias our reported findings.

We assume that any clinical measure can be inherently biased by measurement errors, laboratory techniques, environmental factors, as well as natural biological variability, homeostasis, and/or daily cycles. Although a single accurate snapshot in time can be indicative, a patient’s history is the key to predicting the future, and no other means but a clinical interview allows for gathering a patient’s history in a de novo PD patient. We propose four simple questions about age of onset, history of stroke, fainting, and vocalization during dreams as a quick preliminary screening that can take a fraction of the time required to perform the MoCA or battery of neuropsychological tests. A more comprehensive examination could be conducted if the model shows a considerable risk for future cognitive decline. Since the model underwent development and cross-validation testing on the PPMI dataset and blind validation on independent BIO-PD data, we assume sufficient generalizability of our findings^52^. Given that we report likelihood ratios, future studies could refine our estimates to enhance their applicability.

To conclude, the presented study underlined and quantified the importance of the patient’s history, neuropsychiatric inventories, autonomic symptoms, and REM sleep behavior disorder for the prognosis of cognitive decline in de novo PD. The questionnaire-based markers showed predictive power comparable to expensive instrumentation markers. More importantly, the data can be gathered by patients themselves via online questionnaires, allowing a large-scale preselection of individuals at risk of cognitive impairment in clinical trials.

## Methods

### Database

**Fig. 3:**
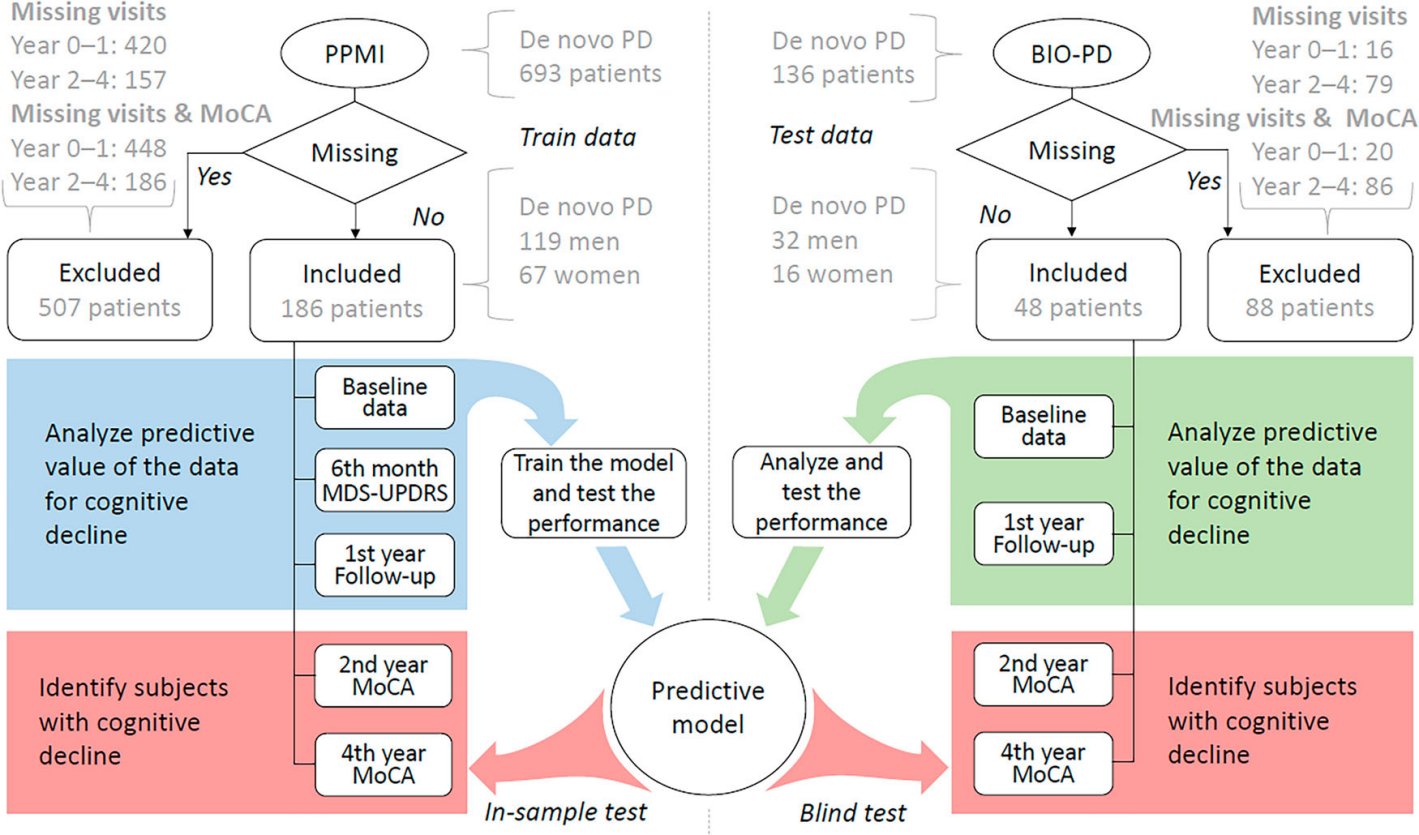
A flow diagram outlining the data analysis and overall experimental concept. Note that the criteria for Missing data were availability of the visits’ data for the baseline and required follow-up visits, including the 6th month, year 1, year 2, and year 4, and availability of the Montreal Cognitive Assessment of baseline visit and year 2 and year 4 follow-ups. Note that the numbers of missing values can overlap between years. Only 42 subjects in PPMI and 37 subjects in BIO-PD had both 2nd and 4th year follow-up missing. PD Parkinson’s disease, PPMI Parkinson’s Progression Marker Initiative database, BIO-PD Biomarkers of Parkinson’s disease database, MoCA Montreal Cognitive Assessment, MDS-UPDRS MDS-Unified Parkinson’s Disease Rating Scale.

The clinical predictors of cognitive decline in newly diagnosed treatment-naive PD patients were investigated on two independent datasets to perform blind validation (please see Fig. 3). First, the Parkinson’s Progression Marker Initiative (PPMI) data collected from patients with PD and healthy controls in 33 sites (USA, Europe, Israel, and Australia) since 2010 with 6-month follow-ups were analyzed. A total of 186 patients with PD enrolled between September 2010 and April 2013 were selected for this study. The criteria for selection were the availability of the visits’ data for the baseline and required follow-up visits, including the 6th month, year 1, year 2, and year 4. Additionally, we excluded all the patients with missing Montreal Cognitive Assessment (MoCA) at baseline visit and year 2 and year 4 follow-ups. Following these criteria, a substantial portion of data was discarded to preserve the integrity, consistency, and comparability of the data while preventing bias introduced by the missing data.

The baseline assessment battery consisted of the University of Pennsylvania Smell Test (UPSIT), the MDS-Unified Parkinson’s Disease Rating Scale (MDS-UPDRS), the Scale for Outcomes in Parkinson’s Disease for Autonomic Symptoms (SCOPA-AUT), State-Trait Anxiety Inventory (STAI-X1 and X2), standard Montreal Cognitive Assessment (MoCA, v1.0), and REM sleep behavior disorder screening questionnaire (RBDSQ). Additionally, we derived dimension subscores of MoCA by summing points of visuospatial/executive, naming, attention, language, abstraction, delayed recall, and orientation domains.

Lateralized MDS-UPDRS III sub-scores for rigidity, akinesia, and tremor were calculated separately for the left and right sides. The rigidity sub-score was calculated by summing the upper and lower limb rigidity scores for each side. The akinesia sub-score was obtained by summing the Finger tapping, Hand Movements, Pronation-supination, toe tapping, and Leg agility scores for each side. The tremor sub-score was calculated by summing the Postural tremor, Kinetic tremor, and rest tremor amplitude scores for the hand and leg on each side. These lateralized motor scores were also combined into a unilateral motor representation by averaging the left and right scores.

The postural instability gait difficulty (PIGD) score was estimated by averaging the following MDS-UPDRS items: Arising from the chair, Gait, Freezing of gait, Postural stability, and Posture^29^. The tremor score was calculated by averaging tremor-related MDS-UPDRS items, including Rest tremor of either face, lips, or chin, all four limbs, and Action/Postural tremor in both arms^53^. PIGD-dominant subjects were identified as Tremor/PIGD ratio ≤ 1.0, whereas the Tremor-dominant subjects were determined by a Tremor/PIGD ratio ≥ 1.5^54^.

Additional data collected included sex, years of education, age of onset, disease duration, body mass index, and medical history (head injury with or without loss of consciousness, family history of PD, anxiety, depression, smoking, diabetes, alcohol intake, epilepsy, encephalitis, and stroke). Follow-up visit at 6th month included MDS-UPDRS, while the 1-year follow-up covered MDS-UPDRS, UPSIT, SCOPA-AUT, STAI-X1 and X2, and RBDSQ. MoCA was assessed at baseline, year 2, and year 4. The PPMI data was used as a training dataset for feature selection and model definition.

Additionally, we included 48 de novo PD patients who were enrolled from February 2015 to August 2018 into the Biomarkers of Parkinson’s Disease (BIO-PD) project for blind validation of the model^55^. Each participant was examined at baseline and follow-ups at years 1, 2, and 4 using the same battery of tests as in the PPMI cohort.

Patients in both PPMI and BIO-PD databases were categorized into subjects without cognitive decline and subjects with cognitive decline. Cognitive decline was defined as a decrease of three or more points in the MoCA score from baseline to the 2-year and/or 4-year follow-up. This threshold was adopted from previous studies by averaging the reported clinically meaningful differences in MoCA over time^56,57^. Having a one-point lower threshold than the recommendation for the Czech MoCA is preferable because participants in PPMI and BIO-PD are younger than those in the cohort studied by Kopecek et al. and are therefore expected to exhibit less MoCA fluctuation^54^. Baseline characteristics of all analyzed subjects are summarized in Table 3.

**Table 3:** Clinical characteristics of patients with Parkinson’s disease included in the study

	PPMI		BIO-PD	
	Patients without cognitive decline (*N* = 137)	Patients with cognitive decline (*N* = 49)	Patients without cognitive decline (*N* = 39)	Patients with cognitive decline (*N* = 9)
Age of onset (years)	58.14 ± 8.97 (31.03–83.15)	62.67 ± 10.27 ** (34.02–81.42)	56.87 ± 11.09 (32.56–76.25)	63.48 ± 14.97 (30.09–80.75)
Disease duration (years)	1.95 ± 2.11 (0.25–20.08)	1.67 ± 1.39 (0.17–6.17)	1.99 ± 2.03 (0.25–11.33)	1.51 ± 1.15 (0.25–4.08)
Men	85/137 (62.04%)	34/49 (69.39%)	24/39 (61.54%)	8/9 (88.89%)
Education (years)	15.47 ± 2.78 (9.00–24.00)	15.10 ± 3.49 (5.00–22.00)	15.59 ± 3.25 (9.00–23.00)	14.44 ± 2.88 (12.00–20.00)
MoCA ***	27.00 ± 2.19 (21.00–30.00)	27.63 ± 1.84 (21.00–30.00)	24.56 ± 3.23 (15.00–30.00)	24.56 ± 3.78 (19.00–30.00)
MDS-UPDRS I	5.25 ± 3.87 (0.00–18.00)	5.27 ± 4.51 (0.00–18.00)	5.44 ± 4.37 (0.00–19.00)	5.22 ± 3.38 (0.00–10.00)
MDS-UPDRS II *	5.41 ± 4.24 (1.00–22.00)	5.67 ± 4.10 (1.00–17.00)	7.08 ± 3.86 (1.00–16.00)	8.22 ± 5.67 (1.00–17.00)
MDS-UPDRS III ***	18.99 ± 8.02 (4.00–41.00)	21.90 ± 8.87 * (6.00–47.00)	27.72 ± 12.26 (6.00–63.00)	28.44 ± 13.01 (10.00–49.00)
SCOPA-AUT	8.99 ± 5.45 (0.00–29.00)	10.46 ± 7.04 (2.00–39.00)	8.85 ± 4.70 (1.00–21.00)	8.67 ± 5.05 (0.00–16.00)
STAI-X1 ***	32.37 ± 9.52 (20.00–64.00)	34.98 ± 10.79 (20.00–61.00)	40.21 ± 9.63 (24.00–65.00)	36.89 ± 8.88 (24.00–51.00)
STAI-X2	40.20 ± 6.49 (31.00–62.00)	42.43 ± 7.71 (32.00–62.00)	39.36 ± 9.48 (21.00–60.00)	35.11 ± 8.80 (23.00–48.00)
RBDSQ *	2.76 ± 2.31 (0.00–10.00)	3.45 ± 2.57 (0.00–10.00)	3.72 ± 3.03 (0.00–10.00)	3.78 ± 2.17 (1.00–7.00)
UPSIT	23.30 ± 8.23 (5.00–39.00)	20.71 ± 6.74 * (5.00–33.00)	23.34 ± 5.84 (14.00–34.00)	18.11 ± 8.25 * (9.00–33.00)
PIGD phenotype	27/137 (19.71%)	10/49 (20.41%)	8/39 (20.51%)	2/9 (22.22%)
Tremor-dominant phenotype	93/137 (67.88%)	32/49 (65.31%)	27/39 (69.23%)	6/9 (66.67%)

Values for Men, PIGD phenotype, and Tremor-dominant phenotype represent total number of positive subjects within the category/total number of subjects in the category (percentual ratio of positive subjects per total number of subjects in the category). Other characteristics were described by mean ± standard deviation (minimum–maximum).

*PPMI* Parkinson’s Progression Marker Initiative database, *BIO-PD* Biomarkers of Parkinson’s disease database, *MoCA* Montreal Cognitive Assessment, *N* total number of subjects per subgroup, *MDS-UPDRS* MDS-Unified Parkinson’s Disease Rating Scale, *STAI-X* State-Trait Anxiety Inventory, *RBDSQ* REM sleep behavior disorder screening questionnaire, *UPSIT* University of Pennsylvania Smell Identification Test, *PIGD* postural instability and gait difficulty.

*p < 0.05, **p < 0.01, ***p < 0.001, probabilities were calculated by *t* test or Wilcoxon rank-sum test for normally and non-normally distributed data, respectively. All probabilities marked in the first column denote significant differences between patients without cognitive decline for PPMI and BIO-PD, all probabilities listed third and/or fifth column denote significant differences between patients with and without cognitive decline within corresponding PPMI or BIO-PD database.

This study was conducted in accordance with the Declaration of Helsinki after approval of the local ethics committees of the participating sites of the PPMI project and from the institutional review board of the General University Hospital in Prague on 24th July 2014 (111/14). All participants gave written informed consent.

### Statistical analysis

The minimum recommended sample size for assessing the accuracy of the prognostic model, assuming an expected sensitivity and specificity of 75%, a type 1 error of 5%, and power of 0.8, is 36 subjects (https://turkjemergmed.com/calculator).

Normality was tested using the Shapiro-Wilk test. Discrete variables were assumed to be non-normally distributed. Normally distributed variables were compared via *t* test, while non-normal data were analyzed via the Wilcoxon rank sum test. Categorical associations were tested via Fisher’s exact test. The correlation of normally and non-normally distributed variables was analyzed via Pearson’s and Spearman’s coefficients, respectively. The threshold of significance was set at *p* < 0.05.

The statistical analyzes are intended to assess whether descriptors show trends for the prediction of cognitive decline in two independent datasets. Since rejecting hypotheses was preferable for determining consistent predictors in both datasets, family-wise error rate was not controlled to avoid inflating type II error. Missing values were removed before statistical comparisons.

For baseline predictor and analyzes of disease progression, MoCA raw scores were converted to z-scores, accounting for age and education as moderating factors to ensure comparability between PPMI and BIO-PD datasets using English and Czech normative data, respectively^58,59^.

We compared baseline characteristics between subjects with and without cognitive decline and analyzed changes in MDS-UPDRS scores from baseline to 6th month (PPMI) and year 1 (PPMI and BIO-PD) to investigate the prognostic values of longitudinal features towards future cognitive decline.

Finally, we applied cut-off thresholds to clinical characteristics, transformed them into binary descriptors, and performed an analysis via likelihood ratios. We then built a prognostic model for future cognitive decline based on likelihood ratio test statistic (LRTS) and demonstrated its practical application. Prior probabilities were estimated within 5-year age brackets using PPMI data.

### Prognostic model

Only clinical variables collected at baseline or initial screening were used to build the predictive model for cognitive decline. Non-binary variables were converted into binary features (presence/absence) using predefined cutoffs. Missing values were treated as absent symptoms, which fits the nature of the analysis while making the analyzed scenario more realistic. The maximum number of missing values per clinical variable was 4 in PPMI and 1 in BIO-PD datasets. Data for smoking were missing more frequently, and treatment of missing values was similar as for other clinical variables.

Cutoff values were adopted from standard clinical thresholds mentioned in the literature for UPSIT, years of education, body mass index, total SCOPA-AUT, MoCA z-score, and total RBDSQ. For other variables, thresholds were optimized through an iterative search. Only variables in line with their underlying hypotheses were accounted for in the model, ensuring that changes in thresholds resulted in predictable performance shifts. We calculated Spearman’s correlation to test if the change of the threshold is consistent with the hypothesis to prevent overfitting of the applied cutoffs (details in Supplementary Data).

The performance of each feature was described using positive and negative likelihood ratios (LRs) in the PPMI dataset. We also evaluated the odds of each symptom presence, calculated as a ratio of symptom-positive subjects and symptom-negative subjects. Only positive LR > 2 (ruling in the cognitive decline) with odds > 0.01 and negative LR < 0.5 (ruling out the cognitive decline) with odds < 0.99 were selected for the model.

Features were combined via a naïve Bayesian classifier with prior probabilities given by the proportion of subjects with cognitive decline. Feature selection was conducted using a model based on prior probability estimated on the whole dataset to avoid feature-selection bias. The final model utilized PD age-of-onset brackets where prior probabilities were estimated within 5-year intervals. Missing values for age of onset were treated with prior probability estimated on the whole dataset.

To simplify the model, only features with a significant contribution to the model performance were retained. We compared the likelihood of a model based on all the features with the likelihood of a model that excludes the tested feature via the likelihood ratio test. The LRTS follows a chi-squared distribution with 1 degree of freedom when only a single feature is being tested. According to the convention, a model excluding the tested feature was preferred over a model with all the features when the LRTS score exceeded the 95% quantile of the reference distribution. This procedure was repeated sequentially until the final model contained only features with significant effect.

To identify redundant features, we performed pairwise comparison using the Jaccard index, defined as the proportion of patients showing both symptoms over those showing at least one. Features sharing > 50% similarity and linked to the same hypothesis were removed.

We performed a repeated random subsampling cross-validation on the PPMI dataset, with 80% of the data used for training, 20% for testing, and 300 repetitions. The final likelihood-ratio model was trained on the overall PPMI and blindly tested on the BIO-PD dataset. The prior probability of future cognitive decline was estimated from a proportion of subjects with cognitive decline within the PPMI dataset. The likelihood ratios of the final model were then used to gradually refine the probability for each symptom present. Each patient was labeled as positive when his or her posterior probability of cognitive decline determined by the model was ≥50%. Subjects with a probability < 50% were labeled as negative. We evaluated the performance by comparing the predicted labels against the reference via sensitivity, specificity, accuracy, and AUC.

## Supplementary information

Supplementary Information

Data Set 1

## Data Availability

Data used in the preparation of this article were obtained on 4 February 2023 from the Parkinson’s Progression Markers Initiative (PPMI) database (www.ppmi-info.org/access-ataspecimens/download-data), RRID:SCR 006431. For up-to-date information on the study, visit www.ppmi-info.org.
