# Supplementary material for: Four questions to predict cognitive decline in de novo Parkinson’s disease
Source: NPJ Parkinsons Dis. 2025 Apr 25;11:91. doi: 10.1038/s41531-025-00958-5 (PMC12022067; doi:10.1038/s41531-025-00958-5)
Supplement: Supplementary file 1 — Supplementary Information [file 41531_2025_958_MOESM1_ESM.docx]

# Supplementary information

Four questions to predict cognitive decline in de novo Parkinson’s disease

Jan Hlavnička^1,2^, MSc, PhD, Josef Mana^1^, MSc, Ondrej Bezdicek^1^, MSc, PhD, Martin Čihák^1^, MSc, Filip Havlík^1^, MSc, Dominik Škrabal^1^, MD, PhD, Tereza Bartošová^1^, MD, Karel Šonka^1^, MD, PhD, Evžen Růžička^1^, MD, PhD, Petr Dušek^1^, MD, PhD

**^1^** Department of Neurology and Centre of Clinical Neuroscience, First Faculty of Medicine, Charles University and General University Hospital, Prague, Czechia

**^2^** Department of Circuit Theory, Faculty of Electrical Engineering, Czech Technical University in Prague, Prague, Czechia

**Correspondence to:** Petr Dušek, MD, PhD, Department of Neurology and Centre of Clinical Neuroscience, First Faculty of Medicine, Charles University in Prague, Kateřinská 30, Prague 2, 128 21, Czech Republic; E-mail: petr.dusek@lf1.cuni.cz

**Keywords:** cognitive decline, predictors of cognitive decline, de novo Parkinson’s disease, prognostic model

**Conflict of Interest:** The authors do not have any relevant disclosures concerning this publication.

**Funding:** Supported by project nr. LX22NPO5107 (MEYS): Financed by European Union – Next Generation EU and the Czech Health Research Council (Grant NU21-04-00535).

# Heterogeneity in the progression of cognitive changes

We observed a great heterogeneity in the progression of cognitive changes in multiple visits. A relatively small portion of patients (18% in PPMI and 11% in BIO-PD) showed a decline in the 2^nd^ visit but did return to normal cognition in the 4^th^ visit without crossing the clinical cutoffs of mild cognitive impairment determined by absolute MoCA scores in any of the visit. The MoCA score did fall below 26 points (the originally established cut-off score for MCI) in the 2nd year and rose back to the cut-off in the 4th year in 33% of PPMI subjects and 50% of BIO-PD subjects. The MoCA score of subjects showing a significant decline between baseline and 2nd-year follow-up and/or baseline and 4th-year follow-up was not below 26 points in any of the visits for 18% of PPMI subjects and 11% of BIO-PD subjects. The cognitive fluctuations could be indicative of future development of cognitive impairment but also show a normal intraindividual variability of cognitive performance.^2^ How to define mild cognitive decline remains an open question and may vary based on study design. When defining the fluctuation by testing a clinical threshold of 26 points for MoCA, the number of subjects was considerably larger for both datasets (33% in PPMI and 50% in BIO-PD).

The likely reason for this phenomenon was that the MoCA scores in both databases are distributed near the clinical cutoff, so the inherent random changes in MoCA scores over time propagated to the results. The discrepancies between PPMI and BIO-PD can be accounted for by the complexity of the Czech language, which renders the translated MoCA more difficult.^3^ All the main factors influencing MoCA including language, age, and education can be compensated by normalizing the MoCA scores according to normative data,^3,4^ which could shift the threshold below the current means of both datasets but may not help for developed stages when MoCA decreases approaching the adjusted cut-off. Also, we observed a discrepancy in distribution of normalized MoCA between PPMI where only 1% of patients showed normalized MoCA below -1 z-score and BIO-PD with around 35% patients with MoCA below -1 z-score. We hypothesize that the baseline MoCA could have been slightly decreased due to anxiety from the first visit of newly recruited patients. Interestingly, the subjects with baseline normalized MoCA bellow 1 declined only in 4^th^ year follow-up and only improvement or stable performance was observed at the 2^nd^ year follow-up which might be related to the anxiety-induced decreased MoCA in baseline.

Therefore, we defined the subjects with cognitive decline as the ones showing a significant decrease in MoCA score between baseline and follow-up and not by applying a clinical threshold on absolute MoCA score. Also, we considered any significant decline in cognitive performance as relevant regardless of whether the performance returned to normal in the next session to estimate the future risk in a wide prediction horizon while regarding the subjective development of early cognitive decline.^2,4^ Such information is advantageous because preventative care such as physical exercise or cognitive rehabilitation should start as soon as possible so that patient can benefit from its long-term effects.^6-11^ Though this prediction is more challenging it is also more clinically relevant as the observed variability within and between datasets is reduced.

# Comparison of characteristics in subjects with and without cognitive decline

The effect sizes were calculated as Cohen’s d, z-statistic divided by a squared number of samples, and odds ratio for t-test, rank-sum test, and Fisher’s exact test, respectively. Note that Cohen’s d and z-statistic effect sizes reported as positive values indicate predictive factors while negative values indicate protective factors.

A significant group difference between patients with and without cognitive decline was observed for age (PPMI: p < 0.01, Cohen’d = 0.46), age of onset (PPMI: p < 0.01, Cohen’s d = 0.48), 1^st^ item od MDS-UPDRS I (BIO-PD: p < 0.05, z = 2.24, r = 0.32), 9^th^ item of MDS-UPDRS III (PPMI: p < 0.05, z = 2.05, r = 0.15), 12^th^ item of MDS-UPDRS III (PPMI: p < 0.001, z = 3.34, r = 0.24), 13^th^ item of MDS-UPDRS III (PPMI: p < 0.05, z = 2.08, r = 0.15), total MDS-UPDRS III (PPMI: p < 0.05, z = 2.08, r = 0.15), left akinesia score (PPMI: p < 0.05, z = 2.07, r = 0.15), 6^th^ question of RBDSQ (PPMI: p < 0.001, odds ratio = 4.73), 7^th^ item of SCOPA-AUT (PPMI: p < 0.01, z = 2.78, r = 0.21), 12^th^ item of SCOPA-AUT (BIO-PD: p < 0.001, z = -3.41, r = -0.49), 4^th^ question of STAI-X1 (PPMI: p < 0.05, z = 2.017, r = 0.15), 16^th^ question of STAI-X1 (BIO-PD: p < 0.05, z = 2.26, r = 0.33), 19^th^ question of STAI-X1 (BIO-PD: p < 0.05, z = 2.179, r = 0.31), 20^th^ question of STAI-X1 (PPMI: p < 0.05, z = -2.09, r = -0.15), 9^th^ question of STAI-X2 (PPMI: p < 0.05, z = 2.13, r = 0.16), 19^th^ question of STAI-X2 (PPMI: p < 0.05, z = -2.22, r = -0.16), UPSIT score (PPMI: p < 0.05, z = -2.05, r = -0.15), history of stroke (PPMI: p < 0.05, odds ratio = *Infimum*), MoCA normalized to z-scores (PPMI: p < 0.01, z = 2.70, r = 0.20).

We observed a significant difference between the patients with and without future cognitive decline in change of the 10^th^ item of MDS-UPDRS III (PPMI: p < 0.05, z = 1.97, r = 0.14), 12^th^ item of MDS-UPDRS III (PPMI: p < 0.05, z = 2.22, r = 0.16), 11^th^ item of MDS-UPDRS II (PPMI: p < 0.01, z = 2.61, r = 0.19), and leg rigidity (PPMI: p < 0.05, z = 2.001888, r = 0.146786) within 6 months after baseline and change of 1^st^ item of MDS-UPDRS III (PPMI: p < 0.05, z = -2.00, r = -0.15), 11^th^ item of MDS-UPDRS II (PPMI: p < 0.01, z = 2.91, r = 0.21), 6^th^ item of RBDSQ (PPMI: p < 0.05, odds ratio = 3.79), 7^th^ item of SCOPA-AUT (PPMI: p < 0.05, z = 2.31, r = 0.17), 9^th^ question of STAI-X1 (BIO-PD: p < 0.05, z = -2.26, r = -0.33), 10^th^ question of STAI-X1 (BIO-PD: p < 0.05, z = 2.31, r = 0.34), 11^th^ question of STAI-X1 (PPMI: p < 0.05, z = -2.07, r = -0.15), 20^th^ item of STAI-X1 (PPMI: p < 0.05, z = -2.27, r = -0.17), and 16^th^ question of STAI-X2 (BIO-PD: p < 0.05, z = 2.03, r = 0.30) within 1^st^ year after baseline.

# Applicability of the prognostic model

The performance on the PPMI data (in-sample AUC = 82%) was not considerably different from the literature utilizing a similar PPMI dataset and a more complex model with a greater palette of features (cross-validated AUC = 80%).^12^ Noteworthy, the PPMI dataset was used only for the training and the prediction horizon included also 4^th^ year follow-up, so the comparability is reduced.^12^ The proposed simple model utilizing clinical questionnaires that offers transparency and flexibility, so the clinician can update the posterior probability based on new available information and bring an actionable insight into patients’ prognosis even when the battery of questionnaires is incomplete. The possibility to adjust the decision threshold towards increased recall (sensitivity ~90%) while keeping the accuracy sufficiently high makes this approach an ideal tool to be built upon. This is advantageous since clinical determination showed a high specificity and low sensitivity in previous research.^13^ The proposed method demonstrates good accuracy; however, we recommend using it primarily as a screening tool, followed by a more detailed cognitive assessment with objective tests, based on previous research.^13^ Although we found that a change in a wide variety of features can be linked to future cognitive decline, the findings were inconsistent between datasets and the vast majority of characteristics would not pass correction for multiple comparisons. Even updating the model with new data from follow-up in year 1 did not improve performance. Nevertheless, the model proved to be applicable for rapid preliminary screening for cognitive decline in de novo PD patients.

|  |  | PPMI | | | BIO-PD | | |
| --- | --- | --- | --- | --- | --- | --- | --- |
|  | Cut-off | Accuracy | Sensitivity | Specificity | Accuracy | Sensitivity | Specificity |
| Baseline features | p > 0.5 | 80 % | 63 % | 85 % | 73 % | 56 % | 77 % |
|  | p > 0.3 | 66 % | 90 % | 58 % | 58 % | 89 % | 51 % |
| Baseline features and follow-up | p > 0.5 | 71 % | 69 % | 72 % | 67 % | 56 % | 69 % |
|  | p > 0.3 | 60 % | 92 % | 48 % | 54 % | 89 % | 46 % |
| Features from questionnaires | p > 0.5 | 79 % | 61 % | 85 % | 73 % | 56 % | 77 % |
|  | p > 0.3 | 66 % | 90 % | 58 % | 58 % | 89 % | 51 % |
| Final simplified model | p > 0.5 | 78 % | 45 % | 91 % | 77 % | 56 % | 82 % |
|  | p > 0.3 | 68 % | 80 % | 64 % | 60 % | 89 % | 53 % |
| Final simplified model with age brackets | p > 0.5 | 78 % | 43 % | 91 % | 77 % | 56 % | 82 % |
| calculated on both PPMI and BIO-PD | p > 0.3 | 69 % | 78 % | 66 % | 63 % | 89 % | 56 % |
| Supplementary Table S1: A performance of various subsets of features for both PPMI and BIO-PD datasets and probability thresholds listed in the “Cut-off” column. Prior probabilities within age brackets were calculated on PPMI data unless indicated otherwise.  BIO-PD = Biomarkers of Parkinson's Disease, PPMI = Parkinson Progression Markers Initiative | | | | | | | |

# References

1. Lewis, F., Butler, A. & Gilbert, L. A unified approach to model selection using the likelihood ratio test. *Methods Ecol. Evol.* **2**, 155-162 (2011).
2. Aarsland, D. et al. Parkinson disease-associated cognitive impairment. *Nat. Rev. Dis. Primers* **7**, 47 (2021).
3. Kopecek, M. et al. Montreal cognitive assessment (MoCA): Normative data for old and very old Czech adults. *Appl. Neuropsychol. Adult* **24**, 23-29 (2017).
4. Rossetti, H. C., Lacritz, L. H., Cullum, C. M. & Weiner, M. F. Normative data for the Montreal Cognitive Assessment (MoCA) in a population-based sample. *Neurology* **77**, 1272-1275 (2011).
5. Feeney, J. et al. Measurement error, reliability, and minimum detectable change in the Mini-Mental State Examination, Montreal Cognitive Assessment, and Color Trails Test among community living middle-aged and older adults. *J. Alzheimers Dis.* **53**, 1107-1114 (2016).
6. Tsukita, K., Sakamaki-Tsukita, H. & Takahashi, R. Long-term effect of regular physical activity and exercise habits in patients with early Parkinson disease. *Neurology* **98**, e859-e871 (2022).
7. Ahlskog, J. E. Does vigorous exercise have a neuroprotective effect in Parkinson disease?. *Neurology* **77**, 288-294 (2011).
8. Schenkman, M. et al. Effect of high-intensity treadmill exercise on motor symptoms in patients with de novo Parkinson disease: a phase 2 randomized clinical trial. *JAMA Neurol.* **75**, 219-226 (2018).
9. van der Kolk, N. M. et al. Effectiveness of home-based and remotely supervised aerobic exercise in Parkinson's disease: a double-blind, randomised controlled trial. *Lancet Neurol.* **18**, 998-1008 (2019).
10. Mak, M. K., Wong-Yu, I. S., Shen, X. & Chung, C. L. Long-term effects of exercise and physical therapy in people with Parkinson disease. *Nat. Rev. Neurol.* **13**, 689-703 (2017).
11. Díez-Cirarda, M. et al. Long-term effects of cognitive rehabilitation on brain, functional outcome and cognition in Parkinson's disease. *Eur. J. Neurol.* **25**, 5-12 (2018).
12. Schrag, A., Siddiqui, U. F., Anastasiou, Z., Weintraub, D. & Schott, J. M. Clinical variables and biomarkers in prediction of cognitive impairment in patients with newly diagnosed Parkinson's disease: a cohort study. *Lancet Neurol.* **16**, 66-75 (2017).
13. Wyman-Chick KA, Martin PK, Barrett MJ, Manning CA, Sperling SA. Diagnostic accuracy and confidence in the clinical detection of cognitive impairment in early-stage Parkinson’s disease. *J Geriatr Psychiatry Neurol*. **30**:178–183 (2017).
